# Medical students’ motivation and academic performance: the mediating roles of self-efficacy and learning engagement

**DOI:** 10.1080/10872981.2020.1742964

**Published:** 2020-03-17

**Authors:** Hongbin Wu, Shan Li, Juan Zheng, Jianru Guo

**Affiliations:** a Institute of Medical Education/National Center for Health Professions Education Development, Peking University, Beijing, China; b Department of Educational and Counselling Psychology, McGill University, Montreal, Canada; c Graduate School of Education, Peking University, Beijing, China

**Keywords:** Intrinsic motivation, extrinsic motivation, self-efficacy, learning engagement, academic performance

## Abstract

**Background**: Motivation matters in medical students’ academic performance. However, few studies have specifically examined how motivation and external environmental factors (e.g., institutions) affect medical students’ performance with large-scale data sets. The roles of self-efficacy and learning engagement in the mechanisms that govern how motivation affects academic performance are still unclear.

**Objective**: This study aims to advance a comprehensive understanding about the relationships between medical students’ motivation, self-efficacy, learning engagement, and academic performance in a nationwide survey, taking students’ demographic factors and sociocultural environments into consideration.

**Design**: We collected data from 1930 medical students in China. We probed the relations between studying variables. We then performed structural equation model (SEM) analysis to examine the mediating roles of self-efficacy and learning engagement on the relationship between motivation and academic performance. We further carried out multiple-group SEM analyses to compare differences between males and females, and between students in key universities and colleges (KUCs) and non-key universities and colleges (NKUCs).

**Results**: Medical students in KUCs demonstrated significantly higher intrinsic motivation, better academic performance and lower extrinsic motivation than those in NKUCs. Male students reported higher intrinsic motivation but surprisingly lower academic performance than females. The total effect of intrinsic motivation on academic performance was larger than that of extrinsic motivation. There were significant indirect effects of either intrinsic or extrinsic motivation on academic performance through learning engagement. Besides, both intrinsic motivation and extrinsic motivation predicted self-efficacy; however, the direct effect of self-efficacy on academic performance was not significant.

**Conclusions**: This study provided researchers with a holistic picture of students’ types of motivation in relation to academic performance. Findings from this study can help in rethinking the role of self-efficacy in medicine, in finding more effective interventions for promoting medical students’ levels of motivation, and in developing motivation-related counselling methods for different groups of medical students.

## Introduction

Motivation matters in medical students’ academic performance due to the highly intensive nature of medicine programs. For example, following a specifically defined path to become a doctor requires carrying out clinical work along with school courses [1,2]. The types of motivation may vary, but they can be generally classified into two categories. One category is intrinsic motivation (e.g., being interested in becoming a doctor, or in pursuing the intellectual challenges of medical science). The other type of motivation is extrinsic, or outcome-oriented, for example being motivated to earn a good salary as a medical professional [3]. Beyond the two categories of motivation, self-efficacy has also received wide attention from medical researchers. Specifically, self-efficacy is an individual’s subjective evaluation of his or her ability to complete a certain task. [4] In achievement-oriented educational settings, self-efficacy concerns a student’s perceived confidence in achieving certain goals. The sense of self-efficacy helps to determine what choices students make, how much mental effort they invest and how long they persist in a task [5]. However, few studies have specifically examined how different motivational components simultaneously affect medical students’ performance with a large sample size [6,7]. Moreover, motivation is a joint product of an individual’s personality and his/her external environment, which suggests medical students’ motivation should be examined in a way that considers both their personal characteristics and the external constraints or situations they face [8]. The purpose of our research was to shed light on the mechanisms that govern how different types of motivation (i.e., intrinsic motivation, extrinsic motivation, and self-efficacy) affect learning engagement and performance in medical education using large-scale data sets, with appropriate consideration of students’ demographic factors and external environmental factors such as the ranking of educational institutions.

Regarding students’ demographic factors, we take student gender into consideration, considering that previous researchers have reported mixed or contradictory findings about gender differences in students’ intrinsic and extrinsic motives [9]. Examining the differences in medical students’ motives by gender may offer promising insights for reducing gender disparities in academic performance and career excellence. Moreover, the extant literature suggests that students’ motivation tend to change over the years of schooling. [7,10] Taking account of the years of curriculum can make the results more comprehensive and convincing.

With respect to external environmental factors, we take the ranking category of educational institution, type of college admission, and home location into account. It is noteworthy that there are two ranking categories of educational institutions in China. The ranking category of key universities and colleges (KUC) includes universities and colleges participating in Project 985 and Project 211 in China. The Chinese government launched these two projects to promote the development of high-quality education. A total of 112 universities and colleges (from a pool of more than 2,000 universities and colleges in China) are recognized as participants in Project 985 and Project 211. These universities and colleges are targeted as national priorities for the implementation of ground-breaking education methods to enable world-class innovation. Therefore, these 112 universities and colleges are highly selective compared with other non-key universities and colleges (NKUC). Students are admitted to KUCs and NKUCs based on a standardized test of the National College Entrance Examination, an academic examination held annually in China. We consider this distinction (KUC vs NKUC) to be a crucial factor, which has never before been investigated in studies of medical students. It is also important to mention that students have several ranked options (a list of ordered preferences) when applying for intended educational institutions and programs in China. For example, an individual can choose a medicine-related major as his/her first choice, and pick chemistry as a second choice. Therefore, we also choose to track the participants’ types of admission, i.e., whether or not they choose medicine as their choice. The choice on students’ part reflects their motivational tendencies to some extent. Finally, we feel that it is relevant to consider the students’ family economic status, which can be broadly indicated by their home locations (i.e., rural areas vs. urban areas).

In sum, we designed the study to address the following three research questions: (1) Do students’ demographic factors (i.e., gender and year of curriculum) and their external environments (i.e., the ranking category of students’ learning institutions, type of admission, and home location) have an influence on their intrinsic motivation (IM) and extrinsic motivation (EM) in medical disciplines? (2) Do medical students with different demographic profiles differ in their intrinsic motivation (IM), extrinsic motivation (EM), self-efficacy (SE), learning engagement (LE), and academic performance (AP)? (3) What are the relationships between IM, EM, SE, LE and AP? How do different types of motivation (i.e., IM, EM, and SE) affect LE and AP? Can any differences in the interaction between the studying variables be observed in distinct population groups?

A number of studies have demonstrated that both intrinsic and extrinsic types of motivation influence a student’s self-efficacy, and self-efficacy has a positive effect on academic performance [3,11,12]. Therefore, we hypothesize that self-efficacy mediates the relationship between intrinsic/extrinsic motivation and academic performance. In addition, researchers have shown that both intrinsic and extrinsic motivation are positively related to students’ levels of engagement in learning, and that engagement is a crucial factor in predicting academic performance [11]. We postulate that learning engagement has also a mediating effect on the relationship between intrinsic/extrinsic motivation and students’ academic performance.

## Method

### Sample

Using a stratified sampling method, we invited students from years 1 to 4 in 10 universities and colleges in China to participate in our research project through an electronic questionnaire, during May and June of 2014. This study was granted an exemption from requiring ethics approval by the Institutional Review Board of Peking University because the survey was anonymous and did not include sensitive questions. An introduction about the survey was provided on the first page of the questionnaire, including aims, the main contents of this survey and promise to keep the data anonymous and confidential. The sampled students had the right to withdraw at any time. Specifically, 2320 medical students were invited to complete the survey, with a response rate was 83.2% (1930). The information regarding students’ academic performance was provided by their respective institutions.

### Measurement

In addition to collecting students’ demographic information, the electronic survey consists of three subscales, including the *Enrolment Motivation Scale*, the modified *Self-efficacy Scale* [13] and the *Learning Engagement Scale*. The three subscales were designed to measure students intrinsic/extrinsic motivation, self-efficacy, and learning engagement, respectively. The items for each subscale were shown in Table 1. In particular, the *Enrolment Motivation Scale* was adapted from the *Academic Motivation Scale (AMS)*, [14] while the *Learning Engagement Scale* was adapted from the *Utrecht Work Engagement Scale (UWES)–Student* [15]. Furthermore, the AMS was developed to assess university students’ intrinsic motivation, extrinsic motivation, and amotivation toward education. The developers contended that the AMS was cross-culturally effective. [14] In terms of the UWES-Student, it measures students’ learning engagement from three aspects, i.e., vigor, absorption and dedication [15].

**Table 1.** Exploratory factor analysis

Scales	Items	Component		KMO
Enrollment motivation	Items	IM	EM	
	I maintain good academic records in natural sciences subjects such as biology in high school	.784	.086	0.73
	I have a strong interest in medicine.	.881	.070	
	I am confident I can succeed in the medicine filed.	.789	.219	
	I believe medicine improves my career prospects.	.295	.720	
	My family or friends strongly encourage or require me to choose medicine.	−.105	.788	
	I anticipate that I can get a good salary in future.	.232	.761	
Self-efficacy	Items	SE		
	If I try my best, I can always solve the problems.	.856		0.72
	When I meet with difficulties, I can usually think of some ways to deal with them.	.886		
	I can calmly face the difficulties, because I trust in my ability	.860		
	Items	LE		
Learning engagement	I feel vigorous and energetic when pursuing content knowledge about medicine.	.878		0.72
	Learning medicine has inspired me.	.910		
	Sometimes I am so involved in my learning that I forget everything around me.	.857		

IM = Intrinsic Motivation, EM = Extrinsic Motivation, SE = Self-efficacy, LE = Learning Engagement, KMO = the result of Kaiser-Meyer-Olkin analysis. Principal component factor extraction with varimax rotation for Enrollment Motivation Scale, and principal component factor extraction for Self-efficacy Scale and Learning engagement Scale. A total of the variance of the three scales were 65.8%, 75.2% and 77.8% respectively.

We conducted principal components analyses to assess the validity and reliability for each of the scales. To assess the appropriateness of using principal components analysis in this study, the Kaiser-Meyer-Olkin test was first performed, which yielded an index of 0.73 on the *Enrolment Motivation Scale* and 0.72 on both the *Self-efficacy Scale* and the *Learning Engagement Scale*. The results from the Bartlett’s test of sphericity for the three subscales were all significant (*p* = 0.000), which allowed us to identify the factor model by using the exploratory factor analysis approach. The summary of results from the factor analysis for the six items of the *Enrolment Motivation Scale* are reported in Table 1. Two factors emerged which accounted for 65.8% of the variance. Factor 1, which involved the first three items, represented intrinsic motivation (IM) with the factor coefficients all larger than 0.7. Factor 2 consisted of the last three items (factor coefficients > 0.7) and this factor was identified as extrinsic motivation (EM). For the *Self-efficacy Scale*, only one factor emerged as expected, which accounted for a total of 75.2% of the variance. Moreover, one factor emerged in the *Learning Engagement Scale* which accounted for 77.8% of the variance. We used the standardized factor scores of the variables (IM, EM, SE, and LE) generated from exploratory factor analysis to conduct further evaluation, because the resulting factor scores were uncorrelated which could improve the accuracy of our follow-up analyses [16].

In terms of the reliability of the three scales (i.e., the *Enrolment Motivation Scale*, the *Self-efficacy Scale* and the *Learning Engagement Scale*), we checked the values of the Cronbach’s alpha coefficients. We found that the internal consistency reliability measure of the *Enrolment Motivation Scale* had a Cronbach’s alpha coefficient of 0.72, which was acceptable. The Cronbach’s alpha coefficients of IM and EM were 0.78 and 0.75, respectively. For the *Self-efficacy Scale* and the *Learning Engagement Scale*, the Cronbach’s alpha coefficients were 0.84 and 0.86, respectively, which indicated that both scales had good reliability.

Students’ academic performance was indicated by their most up-to-date cumulative grade means provided by their institutions. The calculation of cumulative grade means was the same as the calculation of CGPA (cumulative grade points average) but without transforming students’ raw grades into grade points. The mean and standard deviation of AP were 77.95 (the total score was 100) and 10.42, respectively.

### Data analysis

To address our first research question, we performed multivariate regression analyses to determine whether students’ demographic factors (i.e., gender and year of curriculum) and their external environments (i.e., the ranking category of students’ learning institutions, type of admission, and home location) affect their intrinsic motivation and extrinsic motivation toward medicine. We took students’ demographics and external environmental factors as a set of independent variables and took either IM or EM as the dependent variable. To answer the second research question, a series of unpaired *t*-tests were performed to compare the differences in the students’ levels of IM, EM, SE, LE and AP among different population groups. For the third research question, we used Pearson correlation to explore the relationships between the studying variables. SEM (structural equation modelling) analysis was then carried out using the AMOS software. Furthermore, multiple-group SEM analyses were conducted to compare differences between males and females, and students in KUCs and NKUCs. The indices used for estimating the goodness-of-fit of SEM analyses were the chi-square (> 0.05), the comparative fit index (CFI > 0.9), the normed fit index (NFI > 0.9), and the root mean square error of approximation (RMSEA < 0.05).

## Results

### Respondents

There were 573 males (29.7%) and 1,357 females (70.3%) in our sample. The gender distribution was close to the gender ratio of the entire medical student population at China Medical Universities, which was 60.1% females versus 39.9% male students [17]. Also in this sample, 36.7% of the students were from key universities and colleges, and 67.6% choosen medicine as their first-choice major. Moreover, more than half of the students (55.9%) were from rural China, suggesting that their families had a relatively low economic status compared to those from urban areas. Our sample size satisfied the rules of having at least 200 participants, and of having at least 20 subjects for every variable in structural equation model [18]. A summary of the respondent characteristics is presented in Table 2.

**Table 2.** Sample distribution and characteristics of the individual respondents

Variables	N (% of 1,930)
Gender	
Male	573 (29.7)
Female	1,357 (70.3)
Grade	
Freshmen (Year 1)	502 (26.0)
Sophomores (Year 2)	590 (30.6)
Juniors (Year 3)	357 (18.5)
Seniors (Year 4)	481 (24.9)
Method of admission	
Medicine as the first choice (FC)	1,304 (67.6)
Medicine not the first choice (NFC)	626 (32.4)
Types of universities and colleges	
Key universities and colleges (KUC)	702 (36.7)
Non-key universities and colleges (NKUC)	1,228 (63.6)
Home location	
Rural areas	1,078 (55.9)
Urban areas	852 (44.1)

### The characteristics of medical students, motivation and academic performance

Results from multivariate regression analyses showed that the type of university or college (KUC vs. NKUC) significantly positively predicted students’ intrinsic motivation (r = 0.22, *p* = 0.000) and negatively predicted their extrinsic motivation (r = −0.19, *p* = 0.000). However, the variables of gender, grade (i.e., year of curriculum), method of admission, and home location were not significant predictors for either intrinsic motivation or extrinsic motivation.

In addition, we performed a series of unpaired t-tests to assess how students from KUCs differed from those attending NKUCs in terms of IM, EM, SE, LE and AP (see Table 3). We also compared the differences between male and female students, as gender is always a research interest that can inform practice [1,19]. We found that the male students had significantly higher IM, but lower AP than the female students. However, there was no significant difference in EM or SE scores between male and female students. As for the type of university or college, we found that students in KUCs had significantly higher IM and AP, but significantly lower EM than students in NKUCs. There were no significant differences in SE and LE scores between the students in KUCs and those in NKUCs.

**Table 3.** Results of t tests

	Female (1,357)		Male (573)				
Variables	M	SD	M	SD	Difference	*t*	*p*
IM	−0.04	0.99	0.08	1.02	−0.12	−2.41	0.02
EM	0.00	0.99	0.01	1.02	−0.01	−0.22	0.83
SE	0.01	0.98	−0.03	1.06	0.04	0.87	0.39
LE	−0.03	0.98	0.07	1.05	−0.09	−1.87	0.06
AP	78.44	10.24	76.81	10.75	1.63	3.15	0.002
	KUC (702)		NKUC (1,228)				
	M	SD	M	SD	Difference	*t*	*p*
IM	0.15	1.01	−0.09	0.99	0.24	5.15	0.00
EM	−0.11	1.11	0.07	0.93	−0.18	−3.81	0.00
SE	0.02	1.04	−0.01	0.98	0.03	0.70	0.48
LE	−0.04	1.05	0.02	0.97	−0.07	−1.37	0.07
AP	78.83	12.90	77.46	8.65	1.37	2.78	0.005

IM = Intrinsic Motivation, EM = Extrinsic Motivation, SE = Self-efficacy, LE = Learning Engagement, AP = Academic Performance. KUC, Key universities and colleges; NKUC, Non-key universities and colleges. M = mean, SD = standard deviation.

### The mediating roles of self-efficacy and learning engagement

The Pearson correlations between the different variables were as follows (see Table 4). IM was significantly and positively correlated with SE, LE and AP. EM was significantly and positively correlated with SE and LE, but not significantly or positively correlated with AP. It was noteworthy that the correlation coefficient between EM and LE (r = 0.18, *p* < 0.01) was distinctly smaller than that between IM and LE (r = 0.42, *p* < 0.01). Moreover, the results showed that SE was significantly correlated with LE (r = 0.60, *p* < 0.01). In addition, both SE and LE had significant correlations with AP, and those correlation coefficients were 0.12 and 0.14, respectively. These correlations formed the basis for further analysis.

**Table 4.** Pearson correlation between the variables (N = 1,930)

	IM	EM	SE	LE
EM	-	-		
SE	0.34**	0.21**	-	
LE	.42**	0.18**	0.60**	-
AP	0.11**	0.002	0.12**	0.14**

IM = intrinsic motivation, EM = extrinsic motivation, SE = self-efficacy, LE = learning engagement, AP = academic performance. ** *p* < 0.01.

We then performed structural equation model analyses for the whole sample, and separately for the two models of multiple-group analysis (i.e., for groups by gender and type of university or college). The results showed that each model fit well with the data. Table 5 shows an overview of goodness-of-fit measures of the three models.

**Table 5.** Overview of goodness-of-fit measures for structural equation modeling

Models	df	χ2	P (>0.05)	CFI (>0.9)	NFI (>0.9)	TLI (>0.9)	RMSEA (<0.05)
Initial Model (the whole sample)	3	7.78	0.08	1.00	1.00	0.99	0.03
Model for Multiple-group analysis (Gender)	6	15.13	0.19	0.99	0.99	0.97	0.03
Model for Multiple-group analysis (Type of universities and colleges)	6	5.46	0.47	1.00	1.00	0.98	0.00

Note: *df* indicates degrees of freedom; P, Probability level; CFI, comparative fit index; NFI, Normal fit index; TLI, Tucker-Lewin index; RMSEA, Root Mean Square Error of Approximation.

**Figure 1.**
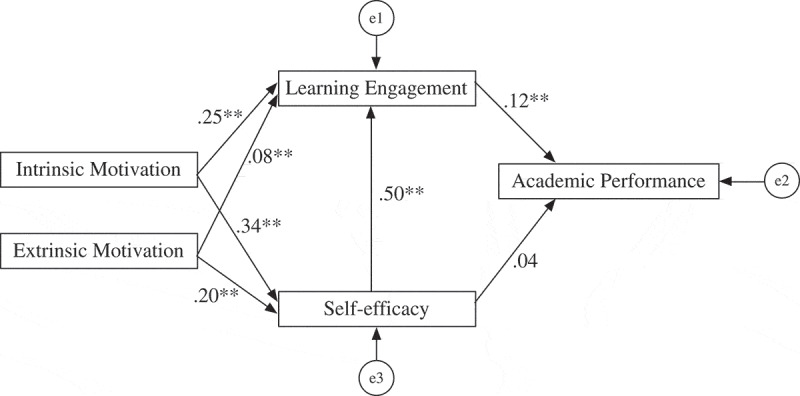
Structural equation model depicting the relationship between motivation, learning engagement, self-efficacy and academic performance. The total effects of intrinsic motivation and extrinsic motivation on academic performance were.06 and .03, respectively. Note: *p < .05, **p < .01

The structural equation model analyses for the whole sample resulted in the model depicted in Figure 1 and Table 6 Part A. These analyses showed the standardized regression weights of the variables. We found that all paths had significant effects except the path linking SE with AP. Moreover, IM had a larger total effect (0.06) than EM (0.03) on AP. IM also showed a greater effect than EM on both LE and SE.

**Table 6.** Results of structural equation model analyses

The whole sample (Part A)	Standardized estimates			S.E.	*p*
IM on SE	0.34			0.02	0.00
EM on SE	0.20			0.02	0.00
IM on LE	0.25			0.02	0.00
EM on LE	0.08			0.02	0.00
SE on LE	0.50			0.02	0.00
LE on AP	0.12			0.30	0.00
SE on AP	0.04			0.30	0.12
Total effect of IM on AP	0.06				
Total effect of EM on AP	0.03				
	Gender		Type of university or college		
Multiple-group (Part B)	Males	Females	KUCs		NKUCs
IM on SE	0.38***	0.32***	0.34***		0.33***
EM on SE	0.24***	0.19***	0.14***		0.25***
IM on LE	0.24***	0.25***	0.17***		0.31***
EM on LE	0.05	0.09***	0.06**		0.09***
SE on LE	0.56***	0.49***	0.59***		0.44***
LE on AP	0.16***	0.11***	0.05		0.19***
SE on AP	0.01	0.05	0.03		0.05
Total effect of IM on AP	0.08	0.06	0.03		0.10
Total effect of EM on AP	0.03	0.03	0.01		0.05

IM = intrinsic motivation, EM = extrinsic motivation, SE = self-efficacy, LE = learning engagement, AP = academic performance. ** *p* < 0.05 and *** *p* < 0.01. Total effects are standardized total effects.

Table 6 Part B shows the results of multiple-group analyses with respect to the factors of gender (male and female) and type of university (KUC or NKUC). We found that IM and EM positively predicted SE, and that SE positively predicted LE. Also, SE had no significant effect on AP, regardless the group concerned. Just as in the initial model (i.e., the whole sample model), IM had a higher total effect on AP than on EM for both the gender and the type of university groups.

## Discussion

This study found that the participating students’ demographic characteristics (i.e., gender, year of curriculum) and the external environmental features (i.e., type of admission, home location) were not determinant factors of their intrinsic and extrinsic motivation towards medicine, except the ranking category of students’ learning institutions. Specifically, medical students who enrolled in KUCs (key universities and colleges) demonstrated significantly higher intrinsic motivation, but lower extrinsic motivation than those pursuing medicine studies in NKUCs (non-key universities and colleges). Considering that few previous studies have taken the types of university or college into consideration, these results make unique contribution to the literature. These findings have the potential to address discrepancies in previous research concerning the motives of medical students. However, it is still unclear whether high level of intrinsic motivation brings students to KUCs, or the academic atmosphere in KUCs fosters the students’ intrinsic motivation. Determining the direction of causation in this relationship can be a promising focus for future research. Moreover, the medical students from KUCs showed significantly higher academic performance than those in NKUCs, as might be expected.

In terms of gender differences, our results suggested that male students reported significantly higher intrinsic motivation, but surprisingly lower levels of academic performance than female students. This finding partially contradicted that of Kusurkar and his colleagues’, as their study indicated that females had higher intrinsic motivation than males in medical education settings [20]. One possible explanation for this contradiction lies in the pervasive cultural expectations for the roles of women and men in medical school and in medicine-related careers. Female students in Asia-Pacific areas are encouraged by their families and by society to pursue careers that are relatively stable, safe and offer high-rewards. Among such careers, medicine is one of the most popular [21]. In line with this expectation, our study clearly indicated that female students had lower levels of intrinsic motivation than males. Another explanation relates to students’ perceptions of career prosperity such as future leadership and workforce equity. Considering that males dominate the medical-related field in China, female students may be reluctant to pursue medicine when thinking of potential discriminations and unpleasant working environments. Therefore, female students had lower level of intrinsic motivation than males.

In terms of the difference-in-differences in performance between male and female students, the findings from this study generally aligned with those of previous studies. First of all, the females in medical schools showed certain personality traits that could be valuable for success in assignments and exams. Specifically, the female physicians and students scored higher on personality traits of helpfulness and relationship consciousness, whereas the males scored higher on traits such as independence and decisiveness [22]. In addition, the female students were found to be very concerned with proving themselves academically [23]. Female medical students commonly have a strong desire to demonstrate their abilities in a competitive atmosphere. It has also been argued that female and male students tend to adopt different approaches towards assessments, with females trying to gain assurance concerning their levels of competency, and male students being more self-confident about their learning progress [24]. Students’ attitude difference towards assessment may be another reason that females outperform male students in academic tests. With respect to the positive relation between self-efficacy and learning engagement, we found no statistically significant differences between male and female students. While this finding is in our expectations, future studies are needed to testify its generalization by gathering new evidence from different student populations.

This study also found that intrinsic motivation was significantly and positively associated with self-efficacy, learning engagement, and academic performance, as has been previously verified by a variety of studies [11,12,25]. Our study also showed that extrinsic motivation was positively related to self-efficacy and learning engagement. However, extrinsic motivation had no significant association with the students’ academic performance, as had been shown in Baker’s study [26]. One explanation for this pattern is that students who particularly focus on the extrinsic consequences of their behavior are generally good at determining objective indictors of their performance, but less capable in making subjective judgements and pursuing a reasoning process [10]. It remains for future research to determine the mechanisms by which extrinsic motivation is related to students’ performances in different genres of assessments. Moreover, extrinsic motivation according to the extant literature does not keep students motivated for long time but it may help them to do a specific job and get a reward. Since we took students’ overall performance into account rather than a specific task, we viewed the result (the lack of relations between extrinsic motivation and academic performance) reasonable. The positive relation between self-efficacy and academic performance observed in our study, and the positive relation between learning engagement and academic performance, have already been widely confirmed, accepted and replicated by other researchers [27,28]. Our study also found a positive relation between the participating students’ levels of learning engagement and self-efficacy, in line with findings by previous studies [12,29].

The results from SEM showed that the total effects of intrinsic motivation on academic performance were larger than the effects of extrinsic motivation on academic performance. It is also important to mention that learning engagement significantly mediates the relations between intrinsic/extrinsic motivation and academic performance. In addition, both intrinsic motivation and extrinsic motivation significantly positively predict self-efficacy. However, the direct effect of self-efficacy on academic performance was not significant. This finding can be explained by the fact that medical studies require students to solve challenging real-life problems (e.g., diagnosing patients and providing appropriate treatments). Therefore, the students cannot acquire sufficient information about their levels of self-efficacy merely from their academic performance. At the same time, however, it is quite possible that a high academic performance in medical studies results from a significant level of mental effort in terms of cognitive activities and emotional engagement. Self-efficacy, as one type of motivational construct, has some effect, but not a determining effect on student performance.

We also did comparisons between groups by gender (male and female) and by type of university (KUC or NKUC) for the aforementioned mediating effects. As in the initial model (i.e., the whole sample model), the direct effect of self-efficacy on academic performance was found to be non-significant for any of the subgroups. In addition, intrinsic motivation had a higher total effect than extrinsic motivation on academic performance, regardless of subgroups. Furthermore, we found that the total effects of both intrinsic and extrinsic motivation on academic performance for male students were larger than those effects for female students. According to the research conducted by D’Lima, Winsler and Kitsantas [9], male students reported greater adherence to performance-oriented goals than females, which may provide one explanation for this phenomenon. With regard to comparisons between groups in the two types of universities, the students from KUCs reported smaller total effects of both intrinsic and extrinsic motivation on academic performance than the students from NKUCs. To the best of our knowledge, no previous studies have examined the relation between types of universities and medical students’ levels of intrinsic motivation or extrinsic motivation. More research is needed to provide further evidence on this relationship.

The findings from this study inform the practice of medical education in several ways. These results can help in rethinking the role of self-efficacy in medicine, in finding more effective interventions for improving medical students’ academic performance, and in developing motivation-related counselling methods for different groups of medical students. Specifically, our study shows that students’ perceived beliefs about their competencies do not reflect their real abilities for achieving goals in medical education. In addition, findings from this study suggest that motivational interventions for students studying medicine in KUCs should be designed towards maintaining these students’ levels of intrinsic motivation, as these students already have relatively high intrinsic motivation. For these students, it would probably be unhelpful to provide them with tangible external rewards, as extrinsic motivation has negative effects on intrinsic motivation [12]. Concerning interventions for students enrolled in NKUCs, appropriate strategies should be introduced to foster their intrinsic motivation. Moreover, our findings suggest that motivation-related interventions (or treatments pertaining to intrinsic motivation and extrinsic motivation) have a greater probability of successfully enabling higher academic performance for male students than for female students.

This study is not without limitations, which should be dealt with in future studies. One significant concern is that data from additional sources should be included and analyzed, as self-reported data can involve social desirability bias, reflecting the students’ propensities to ‘look good’ by providing inaccurate information to researchers. In addition, more detailed research is required to clarify how intrinsic and extrinsic motivation relate to performance in specific courses of study, such as clinical reasoning, principles of biochemistry or fundamentals of neuroscience. Lastly, it would be interesting to examine the influence of multiple motivational components on students’ task performance (e.g., diagnostic accuracy and efficiency) instead of their academic achievements.

## Conclusion

In this study, we explored the relations between self-efficacy, learning engagement, intrinsic motivation, extrinsic motivation, and academic performance by analyzing self-reported data collected from 1,930 medical students and the data provided by their institutions. We also examined the mediating effects of self-efficacy and learning engagement on the relationships between motivation (i.e., intrinsic and extrinsic motivation) and academic performance. We took the effects of students’ demographics (e.g., gender) and external environmental factors (e.g., ranking category of educational institution) into account, which provided new insights on the interplay between the studying variables as they caused differences in student performance. Findings from this study have not only theoretical contributions but also help inform educators, policy makers and administrators about the motivational aspects of learning in medicine.

## Data sharing

The datasets used and/or analyzed during the current study are available from the corresponding author on reasonable request.
